# Molecular Analysis of Sensory Axon Branching Unraveled a cGMP-Dependent Signaling Cascade

**DOI:** 10.3390/ijms19051266

**Published:** 2018-04-24

**Authors:** Alexandre Dumoulin, Gohar Ter-Avetisyan, Hannes Schmidt, Fritz G. Rathjen

**Affiliations:** 1 Max-Delbrück-Center, Robert-Rössle-Str. 10, 13092 Berlin, Germany; alexandre.dumoulin@imls.uzh.ch (A.D.); gohar_ter_avetisyan@hotmail.com (G.T.-A.); 2 Interfaculty Institute of Biochemistry, University of Tübingen, Hoppe-Seyler-Str. 4, 72076 Tübingen, Germany; hannes.schmidt@uni-tuebingen.de

**Keywords:** sensory neurons, axon branching, cGMP signaling, CNP, Npr2, cGKI

## Abstract

Axonal branching is a key process in the establishment of circuit connectivity within the nervous system. Molecular-genetic studies have shown that a specific form of axonal branching—the bifurcation of sensory neurons at the transition zone between the peripheral and the central nervous system—is regulated by a cyclic guanosine monophosphate (cGMP)-dependent signaling cascade which is composed of C-type natriuretic peptide (CNP), the receptor guanylyl cyclase Npr2, and cGMP-dependent protein kinase Iα (cGKIα). In the absence of any one of these components, neurons in dorsal root ganglia (DRG) and cranial sensory ganglia no longer bifurcate, and instead turn in either an ascending or a descending direction. In contrast, collateral axonal branch formation which represents a second type of axonal branch formation is not affected by inactivation of CNP, Npr2, or cGKI. Whereas axon bifurcation was lost in mouse mutants deficient for components of CNP-induced cGMP formation; the absence of the cGMP-degrading enzyme phosphodiesterase 2A had no effect on axon bifurcation. Adult mice that lack sensory axon bifurcation due to the conditional inactivation of Npr2-mediated cGMP signaling in DRG neurons demonstrated an altered shape of sensory axon terminal fields in the spinal cord, indicating that elaborate compensatory mechanisms reorganize neuronal circuits in the absence of bifurcation. On a functional level, these mice showed impaired heat sensation and nociception induced by chemical irritants, whereas responses to cold sensation, mechanical stimulation, and motor coordination are normal. These data point to a critical role of axon bifurcation for the processing of acute pain perception.

## 1. Introduction: Axonal Pathfinding and Branching—Fundamental Processes to Establish Neuronal Circuits

Brain function is critically dependent on the correct wiring of neurons during embryonic and early postnatal stages. Extending axons are tipped by a growth cone; a specialized highly motile structure that recognizes secreted and membrane or extracellular matrix bound molecular guidance cues in its local microenvironment. These guidance factors instruct the migration of the growth cone along the correct path to its target region. It is well accepted that the basic cellular and molecular mechanisms underlying the directional pathfinding decisions of the growth cone are attraction and repulsion caused by graded distributions of guidance cues. In a gradient of a guidance cue, a higher concentration might result in a higher receptor occupancy and activation. This in turn will induce a localized intracellular signaling cascade in the growth cone resulting in growth directed towards higher concentrations of the cue or away from it [1,2,3,4,5]. It is important to emphasize that during growth, axons also arborize to establish contacts with different targets often located in different parts of the nervous system. It is of note that the formation of axonal branches is a hallmark of virtually all neurons in the brain. Axon branching enables an individual neuron to establish contacts and to communicate with different neurons, and therefore increases the complexity of neuronal networks. Both axon pathfinding and branching are basic processes that contribute to the initial pattern of neuronal networks during development. In the mature brain, axon branches might also emerge in response to injury and experience and contribute to plasticity of the nervous system. Indeed, impairments of axonal pathfinding and abnormal branching might result in severe neurological or neurodevelopmental disorders [6,7,8]. Characterization of molecular mechanisms underlying the control of axonal pathfinding and branching is crucial for our understanding of neuronal circuit formation. 

In this review article, we discuss recent progress on cyclic guanosine monophosphate (cGMP) signaling components implicated in axon branching with a specific focus on sensory axons in the developing spinal cord and hindbrain. In vitro studies on axon branching and growth cone behavior, in which cGMP signaling is modulated, are only briefly mentioned here. For a more detailed description of this field the reader is referred to the reviews listed below. These reviews try to provide an integrated view of how axonal branching might work. However, it is important to keep in mind that the functional in vivo significance of these largely in vitro studies for a mechanistic understanding of axonal branching in vivo is less clear and remains to be established [9,10,11,12].

## 2. cGMP Signaling and Growth Cone Steering—Initial Cell Culture Studies

The ubiquitous second messenger cGMP is well known to be involved in a wide range of biological processes [13,14]. A role for cGMP in regulating axonal extension and growth cone steering was initially demonstrated in in vitro cultures 20 years ago by using a growth cone turning assay. In this pioneering work, cGMP modulated growth cone responses to extracellular axon guidance cues [15]. For example, increasing levels of cGMP converted a repulsive signal by semaphorin 3A into attractive extension of axons of *Xenopus* spinal cord neurons and counteracted growth cone collapse [16]. In cell cultures, axonal guidance molecules trigger cytosolic calcium levels in growth cones, which steers direction of growth. Asymmetry of calcium levels is crucial for this process, where high calcium levels on one side of the growth cone promote attraction whereas low levels on the opposite side result in repulsion [17,18,19,20]. Cyclic adenosine monophosphate (cAMP) has also been implicated in modulating asymmetric calcium concentrations across the growth cone via ryanodine receptors [18,21,22]. cGMP has been found to counteract cAMP-mediated axon growth directionality by converting growth cone attraction of netrin-1 to repulsion [21]. Therefore, cGMP as cAMP levels act as switch by modulating calcium channel activities in growth cones to influence the direction of growth [1,23].

The intracellular signals that are elicited by attractive or repulsive axonal guidance molecules provoke changes of the growth cone morphology which require the action of components of the cytoskeleton and the machinery of vesicle trafficking. These processes might function independently or might affect each other [24]. In vitro, there is evidence that exocytosis and endocytosis can occur asymmetrically across the growth cone [25,26], suggesting that membrane trafficking can be instructive for growth cone turning and collateral formation. In cultured dorsal root ganglia (DRG) neurons, microtubules contacting leading edges of the plasma membrane of growth cones induced lamellipodial protrusions by supplying vesicle-associated membrane protein 7 (VAMP7)-positive vesicular membranes. This microtubule-directed membrane transport steers growth cone directionality, and is stimulated by cAMP and inhibited by cGMP [24]. 

It is also conceivable that an increase of local exocytosis might be sufficient to trigger collateral formation of axons, although this has not been studied in detail. Localized exocytosis might regulate cell surface distribution of specific membrane proteins such as receptors for axonal guidance factors. This has been demonstrated for the protein commissureless that regulates axon guidance across the *Drosophila melanogaster* midline by controlling levels of the axonal guidance receptor Robo at the cell surface [27]. Related observations were made on cultured commissural neurons from the chicken spinal cord in which Rab guanine nucleotide dissociation inhibitor (RabGDI) regulated cell surface expression of Robo1 [28]. 

In addition to these studies on growth cone extension, a role for cGMP signaling in the formation of neuronal circuits in vertebrates was also shown by pharmacological manipulations of the soluble guanylyl cyclases that regulate intracellular cGMP levels in neurons [23,29,30,31,32].

## 3. Branching of Sensory Axons within the Spinal Cord—A Versatile System to Characterize Intracellular Signaling Implicated in Axon Branching

**Figure 1:**
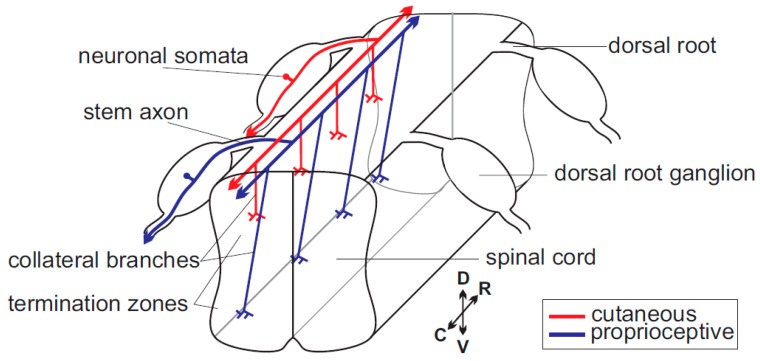
Scheme of the path of sensory axons within the spinal cord. A single cutaneous (red) and single proprioceptive (blue) neuron are highlighted. C, caudal; D, dorsal; R, rostral; and V, ventral.

Despite these fascinating and sophisticated in vitro studies on the role of cGMP signaling in growth cones compiled above, our knowledge of cGMP signaling-related axon pathfinding and branching during developmental stages in the nervous system of vertebrates is still fragmentary. However, a number of recent studies that focused on axon branching have shed light on the involvement of cGMP in neuronal circuit building in vivo. The analysis of DRG neuron axon projections into the spinal cord was instrumental in unravelling the function of cGMP in axon bifurcation—a specific form of axon branching. The pattern of sensory axon branching is relatively simple and stereotyped and therefore appeared suitable for a molecular analysis to characterize branching factors (Figure 1). In contrast, reconstructions of electron microscopic images had shown a much more complex pattern of axonal branches of individual neurons from the rodent brain (for example see neurons in [33,34]). It might be challenging to identify specific molecular signals implicated in shaping the pattern of branches of these cases, assuming that a wide range of intrinsic branch formation programs and external signals are involved in axon branching. 

**Figure 2:**
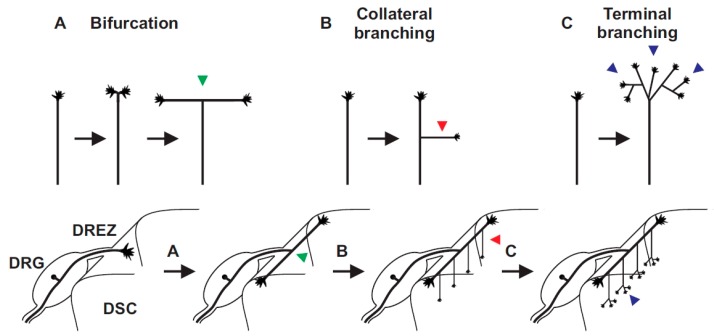
Three branching modes can be deduced from sensory axons in the spinal cord: (**A**) bifurcation (arrow head in green); (**B**) collateral formation from stem axons (arrow head in red); and (**C**) terminal branching in specific layers of the spinal cord (arrow head in blue). DREZ, dorsal root entry zone; DRG, dorsal root ganglia; DSC, dorsal spinal cord.

Sensory axons enter the spinal cord at early embryonic stages at the dorsal root entry zone (DREZ), where they split into ascending and descending arms [35,36,37,38]. These two stem axons grow over several segments along the lateral margin of the cord. Subsequently, axon collaterals branch off from these stem axons (also termed interstitial branching) and grow in ventral direction. Nociceptive and mechanoreceptive collaterals terminate in dorsal layers of the spinal cord, where further terminal branching takes place to establish contacts to dorsal horn neurons. Proprioceptive collaterals extend into ventral parts of the spinal cord including the Clarke column at lower spinal levels and further branch to form synapses on motor neurons or interneurons. Taken together, a relative simple pattern of axon branching is executed by sensory axons within the spinal cord with three major types of branching modes (Figure 2): (A) bifurcation at the DREZ; (B) collateral branching from stem axons; and (C) terminal branching of collaterals in termination zones [39,40]. Images of individual sensory axons when entering the spinal cord at the DREZ indicated that bifurcation is mediated by splitting of the growth cone. In contrast, collateral formation proceeds from the shaft of the two resulting axons. Terminal branching might be regulated by the growth cone or occurs in close distance to the extending growth cone and could be therefore very similar to collateral formation.

**Figure 3:**
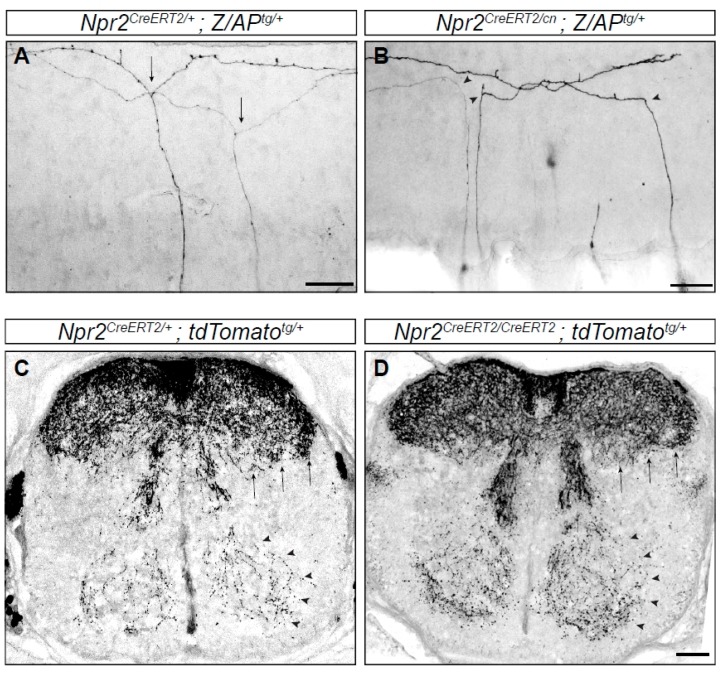
Branching errors of primary sensory neurons in the spinal cord. Individual axons are labeled by a genetic sparse labeling method. (**A**) Two DRG axons from a *Npr2* heterozygous mouse show T-shaped branches (arrows) at the DREZ of the spinal cord. (**B**) In homozygous *Npr2* mutants DRG axons turn in rostral or caudal direction but do not bifurcate (arrow heads). (**C**,**D**) Collaterals are formed in the absence of Npr2 (**D**) as in heterozygotes (**C**) shown in cross sections of the E17.5 spinal cord using a reporter mouse line encoding red fluorescent protein which is activated by tamoxifen injections via Npr2-CreERT2 (causes recombination estrogen ligand-binding domain). Therefore, only Npr2-positive sensory neurons and their axons and collaterals are labeled. However, since the recombination efficiency varies from embryo to embryo due to the variable uptake of tamoxifen and due to the presence of two *Npr2-CreERT2* alleles in the *Npr2* knockout no quantitative conclusions on the amount of collaterals can been drawn from (**C**,**D**). Staining with antibodies to peripherin or TrkA (tropomyosin receptor kinase A) showed that in homozygous *Npr2* mutants collaterals are reduced due to the bifurcation defect [43,45,46]. Arrows point to nociceptive collaterals of deep dorsal horn layers and arrow heads to proprioceptive collaterals. Scale bar in (**A**,**B**), 50 µm and in (**C**,**D**) 100 µm.

It is of importance to note that all sensory axons show more or less the same pattern of branching. This repetitive pattern of arborization is of great advantage for a molecular analysis of branching factors. Moreover, the system is easily accessible to analyze the path of axons by 1,1′-dioctadecyl-3,3,3′3′-tetramethylindocarbocyanine perchlorate (DiI) tracing [41], by antibody staining or by genetic sparse labeling [42,43]. For example, collateral formation of sensory axons is easily accessible for detection by selective antibody staining of cross sections of the spinal cord (e.g., by using antibodies specific for peripherin or tropomyosin receptor kinase A (TrkA)) or by specific mouse reporter lines (see Figure 3C,D). 

## 4. A cGMP Signaling Cascade Regulates Axon Bifurcation but Not Collateral Formation or Terminal Branching

**Figure 4:**
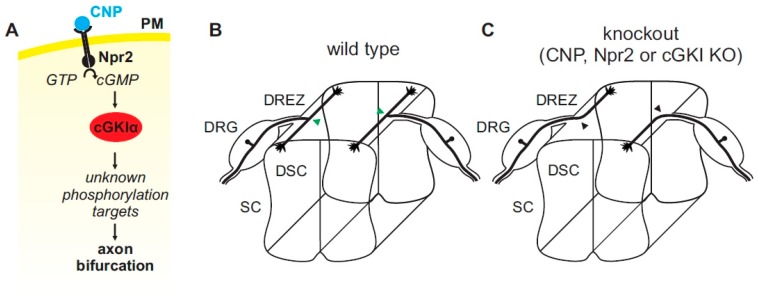
(**A**) A cGMP (cyclic guanosine monophosphate)-dependent signaling cascade composed of CNP, Npr2, and cGKI (cGMP-dependent protein kinase I) is implicated in the bifurcation of sensory axons. In the absence of any of these components sensory axons do not bifurcate when entering the spinal cord or the hindbrain (please compare (**B**,**C**) for the wild type and the knockout situation, respectively). Arrow heads in green (**B**) point to the bifurcation of sensory axons in the wild type and arrow heads in black (**C**) point to bifurcation errors in knockouts of *CNP*, *Npr2* or *cGKI*. The downstream phosphorylation targets of cGKI involved in axon bifurcation have not been defined yet. DRG, dorsal root ganglion; DREZ, dorsal root entry zone; DSC, dorsal spinal cord; SC, spinal cord.

The axonal system described above has enabled the characterization of a cGMP-dependent signaling cascade essential for the bifurcation of sensory axons by applying mouse genetics. This signaling cascade is composed of the ligand C-type natriuretic peptide (CNP), the receptor guanylyl cyclase Npr2 (also designated GC-B or Npr-B) and the cGMP-dependent protein kinase I (cGKI, also known as PKGI (protein kinase G))—a key effector of cGMP signaling cascades [43,44,45,46,47,48]. Two alternatively spliced isoforms of cGKI are known in vertebrates—termed α and β—from which the α-form is expressed in DRG neurons. Upon binding, the ligand CNP activates its homodimeric receptor Npr2 which in turn generates cGMP from guanosine-5’-triphosphate (GTP) by its C-terminal guanylyl cyclase domain. cGMP then stimulates the serine and threonine kinase cGKI. In the absence of any one of these components in mouse knockouts, central axons from DRG neurons no longer bifurcate and instead either turn in a rostral or caudal direction within the spinal cord (Figure 3A,B and Figure 4). Consideration of quantitative data suggest that all subsets of DRG neurons are affected. Consistent with these observations is the timing and pattern of localization of CNP in neurons and precursors of the dorsal spinal cord whereas the related ligands ANP (A-type natriuretic peptide) or BNP (B-type natriuretic peptide) are not expressed [46,48]. Although DRG neurons are extremely heterogeneous [49] Npr2 and cGKI are expressed in all DRG neurons but not in the dorsal horn at early developmental stages [43,44,45]. A critical missing link of the Npr2-mediated cGMP signaling pathway is the characterization of phosphorylation targets of cGKIα in sensory growth cones that mediate axon bifurcation. Such data might provide mechanistic insights into the machinery for bifurcation. Recently, in vitro experiments analysing collateral branching suggested that cGMP signaling regulates microtubule dynamics [50]. 

Subunits of the nitric oxide-sensitive guanylyl cyclases (NO-GCs)—in some in vitro experiments involving the application of pharmacological reagents shown to be involved in growth cone activities [23,47]—are not expressed in embryonic DRG neurons when their axons enter the spinal cord [45]. Two isoforms of NO-GC are known which consist of one α (α1 or α2) and a β subunit (β1). The β1-subunit is the common dimerizing subunit of both NO-GCs. If this subunit is absent in a global mouse knockout NO-induced cGMP signaling is completely eliminated although α subunits are still expressed [51,52]. In the β1 subunit knockout sensory axon bifurcation is normal as well as collateral formation [46]. In addition, the intracellular cGMP level only increased immediately upon application of CNP, but not of ANP, BNP or the NO donor DEA (2-(*N*,*N*-dethylamino)-diazenolate-2-oxide dethylammonium salt)/NO (nitic oxide) in real-time imaging experiments with a genetically encoded fluorescent cGMP sensor in cultivated embryonic mouse DRG neurons [53]. Taken together, it is unlikely that NO-GCs play a role in bifurcation, collateral formation or axon extension in this neuronal system in vivo.

Interestingly, the loss of axon bifurcation in DRG neurons in the absence of cGMP signaling does not affect their ability to form normal collaterals in the spinal cord (Figure 3C,D). Also, the number of collaterals per µm axon segment is not altered, however the total number of collaterals is reduced due to the bifurcation defect and the consequential loss of one half of the longitudinal stem axons [43,45,46]. Additionally, based on antibody staining the superficial layers of the dorsal horn appear to be unaffected by impaired cGMP signaling; suggesting that signaling cascades distinct from Npr2-mediated cGMP signaling regulate collateral formation of sensory axons. These findings also indicate that bifurcation is not a unique case of a common branching process. In fact, it is a specific branching mode that is most likely regulated by the properties of the growth cone. External cues that induce collaterals from the sensory stem axons in the spinal cord have not yet been identified. However, recent progress has been made to characterize an intrinsic factor—MAP7 (Microtubule associated protein 7)—implicated in interstitial branching. In a mouse mutant that expresses a truncated version of MAP7, a promotion of collateral formation was described which was accompanied with an increased pain sensitivity [54]. 

## 5. The CNP/Npr2/cGKI Signaling Cascade Induces Bifurcation of Axons from Three Types of Neurons: DRG, Cranial Sensory Ganglia (CSG), and Mesencephalic Trigeminal Neurons (MTN)

A major question is whether the CNP/Npr2/cGKI signaling cascade is also utilized by other projecting axons to bifurcate during development. Generation of reporter mice of CNP and Npr2 and highly specific antibodies to cGKI allowed the identification of additional axon systems that co-express Npr2 and cGKI and the ligand CNP in the region where bifurcation takes place. These investigations showed that cranial sensory ganglia (CSG) neurons [43] and MTNs (also abbreviated as MesV or Me5) express Npr2 and cGKI, and bifurcate in specific regions of the hindbrain. In the absence of Npr2, CSG axons do not bifurcate anymore in the hindbrain and instead turn in either ascending or descending direction [43]. CSG belong to the peripheral nervous system and transfer sensory information to neurons within the hindbrain. As soon as axons from CSG enter the hindbrain, they generate an ascending and descending branch from which collaterals are generated. In contrast to the DRG neurons of the trunk, a substantial portion of the CSG neurons largely arise from a specialized ectoderm (the sensory placodes) and from neural crest cells [55]. For example, the neurons of the inner ear arise from the otic placode, the nodose placode contributes to the tenth cranial nerve, the vagus nerve, and the large trigeminal ganglion is derived from both ectodermal placodes and neural crest cells [56,57]. 

MTNs are the only primary sensory neurons whose cell somata are located in the central nervous system (CNS) and are, like DRG neurons, pseudo-unipolar. MTN axons initially project from dorsal layers of the mesencephalon ventrally before they pioneer the lateral longitudinal fasciculus to extend further caudally [58,59,60,61]. In the hindbrain, MTN axons form Y-shaped branches [62,63]: one resulting arm leaves the hindbrain and passes through the dorsal root of the trigeminal ganglion (gV) to grow to the jaw, while the other arm of the bifurcation projects to the trigeminal motor nucleus (abbreviated Vmo or Mo5) and to the supratrigeminal nucleus (Vsup) of the hindbrain [64,65]. MTNs innervate spindles of jaw closing muscles (masseter, temporalis) or form mechanoreceptors in the periodontal ligaments [66]. They process proprioceptive information from these mandibular structures and thus are essential in coordinating biting, ingestion, and mastication [67,68,69]. In global *Npr2* knockout mice MTN axons do not split in the hindbrain and either grow to the jaw or to the trigeminal motor nucleus (our unpublished research [70]).

In summary, it appears that axon splitting regulated by CNP/Npr2 signaling via cGKIα is a unique feature of certain primary sensory neurons in vertebrates.

## 6. The Role of Phosphodiesterase 2A and the Scavenger Receptor Npr3 in Sensory Axon Bifurcation

Intracellular cGMP levels are controlled by the rate of its synthesis via guanylyl cyclases and degradation via phosphodiesterases (PDEs) [71,72]. The presence and activity of PDEs might be crucial to control the time of onset of cGMP elevation and to limit the spatial and temporal expansion of the signal. The hydrolysis of cGMP might therefore contribute to the fine-tuning of cGMP signals in sensory axons. mRNAs of a number of the known PDEs which are either activated or inhibited by cGMP, or specifically hydrolyze cGMP, or which are dual substrate enzymes and degrade both cAMP and cGMP are expressed in E12.5 DRGs. Application of selective PDE blockers indicated that only PDE2 specific inhibitors Bay 60-7550 and EHNA (erythro-9-(2-hydroxy-3-nonyl) adenine) caused an increase of the intracellular cGMP levels upon stimulation of cultured embryonic DRG neurons with CNP. Other pharmacological blockers such as vinpocetine (specific for PDE1), milrinone (PDE3), sildenafil (PDE5), or zaprinast (PDE5, 6, 9, 10, and 11) were found not to increase cGMP levels upon CNP stimulation. Therefore, PDE2A is the functionally relevant PDE to hydrolyze CNP-induced cGMP in embryonic DRG neurons. For example, in PDE2A-deficient embryonic DRG, the level of CNP-induced cGMP increased significantly; however, this increase did not perturb the bifurcation, as DRG axons showed normal T-like bifurcations and did not form multiple or ectopic branches [53].

The mRNA of the scavenger receptor Npr3 (also termed Npr-C) is not expressed by DRG neurons but localized in cells—most likely Schwann cells or their precursors—associated with the dorsal roots of the spinal cord [48,53]. While loss-of-function mutations of *Npr3* caused skeletal overgrowth in rodents due to increased levels of CNP in the extracellular space [73,74], on DRG axons Npr3 does not have an important scavenging function since the overall bifurcation process is not disturbed [53]. Taken together, the absence of PDE2A does not interfere with axon bifurcation and the influence of the scavenger receptor Npr3 on sensory axon bifurcation is limited to a minor degree. Therefore axon bifurcation is resilient to high cGMP levels.

## 7. Behavioral Consequences of the Absence of Sensory Axon Bifurcation: Nociception Is Impaired, Whereas Motor Balance and Coordination Is Normal

**Figure 5:**
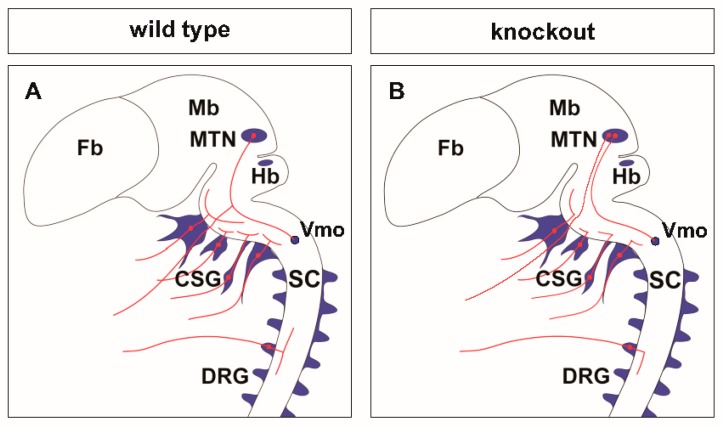
**Scheme of** neuronal circuits with sensory axon bifurcation in the wild type (**A**) or in the absence of bifurcation in *CNP*, *Npr2* or *cGKI* null mutants (**B**). The lack of bifurcation affects the sensory representation of the body in the central nervous system (CNS). CSG, cranial sensory ganglia; DRG, dorsal root ganglia; Fb, forebrain; Hb, hindbrain; Mb, midbrain; MTN, mesencephalic trigeminal neuron; SC, spinal cord; Vmo, trigeminal motor nucleus.

The primary sensory representation of the body within the spinal cord is based on the intricate innervation and branching pattern of axons from DRG neurons. This topographic representation of the soma is of fundamental importance for sensory information processing [75,76]. In the absence of bifurcation, sensory topographic representation is incomplete (Figure 5). To study the functional consequences of the lack of axon bifurcation in the spinal cord in the absence of other phenotypes that may complicate the interpretation of results, *Npr2* and *cGKI* were conditionally inactivated in DRG neurons at early stages using the Cre-driver line *Wnt1-Cre*. In these mice, sensory axon bifurcation is completely lacking as in global knockouts. Surprisingly, in a number of behavioral tests that examine balance and motor coordination (balance beam test, rotarod, staircase assay, food grasping and reaching assay, and walking track analysis) no deficits were observed indicating that despite the absence of sensory axon bifurcation considerable coordination capabilities are maintained in these mice [77]. 

In contrast, loss of axonal bifurcation impairs the rapid response to avoid noxious heat (hot plate test and Hargreaves test), whereas behavioural thresholds and response latencies to cold (acetone-evoked evaporative cooling) or mechanical stimuli (dynamic plantar aesthesiometer) were not affected. Nociception induced by the chemical irritants capsaicin or formalin are impaired by the loss of axonal bifurcation [77]. Consistently, spinal dorsal horn neuron responses to capsaicin were reduced in global *Npr2* and *CNP* knockout mice [45,46]. In addition, a recent study using a constitutive *Npr2* mutant—in which bone growth is reduced—demonstrated deficits in the auditory system [78]. In summary, these data point to a critical role of axonal bifurcation for the processing of pain evoked by heat or chemical stimuli whereas proprioception is more or less normal in the absence of axon bifurcation. 

## 8. Compensatory Mechanisms Alter the Spatial Extension of Receptive Fields in the Spinal Cord in the Absence of Sensory Axon Bifurcation

Sensory information from a large number of afferent axons converges in the spinal cord in nociceptive, mechanoreceptive, or proprioceptive fields. Generation of these distinct and overlapping sensory fields in the spinal cord relies on axon collaterals and terminal branches in specific layers. The influence of a loss of bifurcation on the size or shape of termination fields of afferents was recently visualized by transganglionic transport of fluorescently labelled cholera toxin B (CTB). This work showed not only a quantitative reduction of incoming fibers, but also a change in the pattern in the termination fields—most likely caused by an altered terminal branching [77]. For example, Npr2-deficiency caused an increase of the dorsoventral span of the termination field of digit two of the hind paw whereas the mediolateral extension was narrowed. In principle pre- and postsynaptic mechanisms contribute to the formation of terminal fields. It is likely that the balance between these interacting structures is disordered in the spinal cord of *Npr2* mutants. 

The changes in the termination fields detected by CTB labeling indicate that elaborate compensatory mechanisms are implemented to reorganize neuronal circuits in the absence of bifurcation. The behavioral studies suggest that these compensatory mechanisms might be more active in the proprioceptive than in the nociceptive system.

## 9. CNP/Npr2 Signaling in Human Diseases

CNP and Npr2 are also implicated in the process of endochondral ossification which affects long bone growth. Therefore, biallelic loss-of-function mutations including missense, nonsense, frame-shift mutations, insertions and deletions, and splice site mutations in the human *NPR2* gene result in acromesomelic dysplasia type Maroteaux (AMDM; OMIM602875), a skeletal dysplasia with an extremely short and disproportionate stature [79,80,81]. Moreover, gain-of-function mutations in the human gene resulted in overgrowth [82,83]. Similarly to human patients, constitutive *Npr2*- or *CNP*-deficient mice show dwarfism [84,85,86,87,88]. Whether the absence of CNP/Npr2-mediated cGMP signaling in DRG neurons causes branching errors of sensory axons within the spinal cord in these patients is currently not known. Unfortunately, detailed neurological tests to investigate the occurrence of neurological deficits in AMDM patients are currently lacking. The recent behavioral testing of *Npr2*-deficient mouse mutants might provide a framework for future studies to characterize neurological qualities of human patients with mutations in the *Npr2* gene.

## Abbreviations

AMDM	Acromesomelic dysplasia, Maroteaux type
ANP	Atrial natriuretic peptide
BNP	Brain natriuretic peptide
C	Caudal
cGKI	cGMP-dependent kinase I
CNP	C-type natriuretic peptide
CSG	Cranial sensory ganglia
D	Dorsal
DEA/NO	2-(*N*,*N*-dethylamino)-diazenolate-2-oxide dethylammonium salt
DREZ	Dorsal root entry zone
DRG	Dorsal root ganglia
DSC	Dorsal spinal cord
Fb	Forebrain
gV	Trigeminal ganglion
Hb	Hindbrain
MAP7	Microtubule-associated protein 7
Mb	Midbrain
MTN	Mesencephalic trigeminal neuron
NO	Nitric oxide
NO-GC	Nitric oxide guanylyl cyclase
Npr2	Natriuretic peptide receptor 2
Npr3	Natriuretic peptide receptor 3
PDE	Phosphodiesterase
PM	Plasma membrane
R	Rostral
SC	Spinal cord
V	Ventral
Vmo	Trigeminal motor nucleus
